# BITS2018: the fifteenth annual meeting of the Italian Society of Bioinformatics

**DOI:** 10.1186/s12859-019-3175-9

**Published:** 2019-11-22

**Authors:** Francesca Cordero, Raffaele A. Calogero, Michele Caselle

**Affiliations:** 1 Department of Computer Science, University of Turin, via Pessinetto 12, 10149 Torino, Italy; 2 Department of Molecular Biotechnology and Health Sciences, University of Turin, Via Nizza 52, 10125 Torino, Italy; 3 Department of Physics, University of Turin, Via Pietro Giuria 1, 10126 Torino, Italy

**Keywords:** BITS, Bioinformatics, Meeting of the Italian Society of Bioinformatics

## Abstract

This preface introduces the content of the BioMed Central Bioinformatics journal Supplement related to the 15th annual meeting of the Bioinformatics Italian Society, BITS2018. The Conference was held in Torino, Italy, from June 27th to 29th, 2018.

## BITS, the Italian Society of Bioinformatics

BITS, the Italian Society of Bioinformatics [1], is the largest non-profit italian association of researchers involved in bioinformatics. The goal of BITS is the creation of a multi-disciplinary research community involving Academia, National Research Council and small/medium enterprises.

The environment created during the BITS2018 meeting provided a unique opportunity for all participants to meet, debate with, and get to know the latest activities of the Italian Bioinformatics community.

Since 2004, the BITS society has organized the annual meeting to gather not only the BITS members but all the researchers interested in the bioinformatics field. These events soon became the most important annual meeting for the Italian bioinformatics community, and every year the local organizing committee complements the BITS meeting with workshops and activities centered on the main current hot research topics.

## BITS2018 annual meeting

The BITS2018 meeting was held in Torino, from June 27th to 29th in the Aula Magna of the Cavellerizza Reale of the University of Turin. BITS2018 highlights keynotes talks by excellent scientists in bioinformatics and its applications, oral presentations of the state-of-the-art research in computational biology, and poster session on the latest research progress. The meeting was organized by Francesca Cordero (Department of Computer Science, University of Turin), Raffaele A. Calogero (Department of Molecular Biotechnology and Health Sciences, University of Turin) and Michele Caselle (Department of Physics, University of Turin), with the support of the BITS steering committee. About 180 participants attended the meeting and most of them submitted contributions as oral presentation or poster. After the abstracts evaluation, the BITS2018 Program Committee (see Table 1) selected 29 proposals for oral presentations and 120 posters. The invited speakers were: Theodore C. Goldstein (University of California, San Francisco), Nicola Segata (Centre for Integrative Biology, CIBIO), Mihaela Zavolan (University of Basel) and Stefano Gustincich (Istituto Italiano di Tecnologia - IIT, and Scuola Internazionale Superiore di Studi Avanzati - SISSA). The conference covered a wide range of topics: *Algorithms for Bioinformatics* to *Gene regulation*, *Transcriptomics and epigenomics*, *Protein structure and function*, *Systems Biology*, *Bioimaging* and more. In addition, a special session was devoted to the *Bioinformatic challenge in microbiome research*.

**Table 1:**
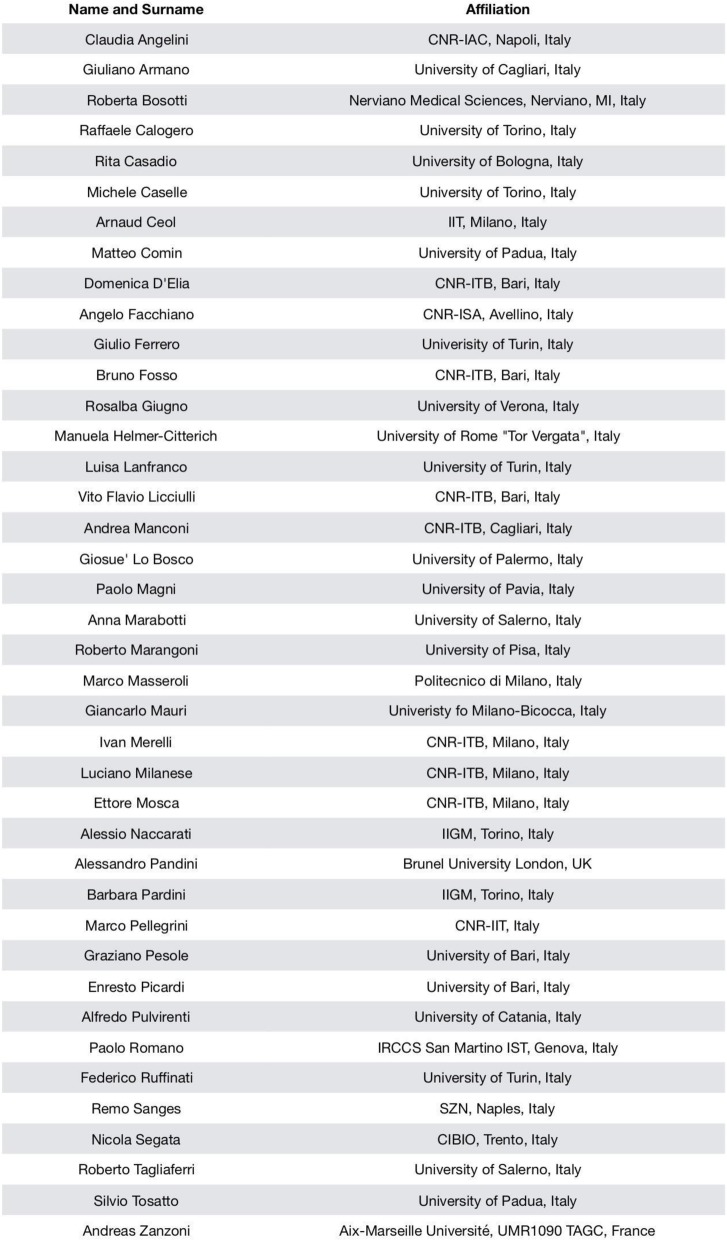
BITS2018 program committee

Four satellites events of BITS2018 were held jointly with the meeting on this 2018 edition. *Docker and Reproducibility*, dedicated to exploiting Docker for reproducibility in bioinformatics analysis; *Single Cell Revolution*, to discuss the opportunities and challenges in Single-Cell Biology; *Precision oncology* to explore the genomics and bioinformatics for precision oncology. Moreover, we organized the *Career Morning* dedicated to Post Docs and PhD students in which career talks were presented by academy professionals and industry researcher to help the graduate students with their career development.

## BITS2018 supplement to BMC bioinformatics journal

All authors of scientific contributions were invited to prepare and submit a manuscript as an extended version of the work presented during the conference. The papers were assigned to an Associated Editor, on the basis of his/her expertise. After editors preliminary evaluation manuscripts were assigned to independent reviewers accordingly to BMC guidelines, avoiding any conflict of interest. At the end of the process, 11 articles (three software papers, one methodology paper and seven research papers) were accepted and included in this supplement. A brief summary of each contribution is reported in the following.

## Alaimo et al. *TACITuS: Transcriptomic Data Collector, Integrator, and Selector on Big Data Platform*

In this paper, the authors introduced TACITuS, a web-based system supporting rapid query access to high-throughput microarray and NGS repositories. The system is equipped with modules capable of managing large files, storing them in a cloud environment and extracting subsets of data in a efficient way. The system also supports the import of data into Galaxy for further analysis.

## Bitar et al. *Genes with human-specific features are primarily involved with brain, immune and metabolic evolution*

The authors proposed a comprehensive study which updated the number of human-specific genes following a critical bibliographic survey. Human-specific genes were functionally assessed providing unique information. The results presented are consistent with environmental changes, such as immune challenges and alterations in diet, as well as neural sophistication, as significant contributors to recent human evolution.

### Dalsass et al. *STRAIN: an R package for multi-locus sequence typing from whole genome sequencing data*

The authors present STRAIN (ST Reduced Assembly IdentificatioN), an R package implementing a hybrid strategy between assembly and mapping of the reads to assign the ST to an isolate starting from its read-sets. STRAIN is designed for single allele typing as well as MLST. Its implementation in R makes allele and ST assignment simple, direct and prompt to be integrated in a wider pipeline of downstream bioinformatics analyses.

### Qian et al. *MetaCon: Unsupervised Clustering of Metagenomic Contigs with Probabilistic k-mers Statistics and Coverage*

The authors present MetaCon a novel tool for unsupervised metagenomic contig binning based on probabilistic k-mers statistics and coverage. MetaCon uses a signature based on k-mers statistics that accounts for the different probability of appearance of a k-mer in different species, also contigs of different length are clustered in two separate phases.

### Shibuya et al. *Better quality score compression through sequence-based quality smoothing*

The authors present YALFF (Yet Another Lossy Fastq Filter), a tool for quality scores compression by smoothing leading to improve compressibility of FASTQ files. Authors used FM-Index to reduce the storage requirements of a dictionary of k-mers and an effective smoothing algorithm to maintain high precision for SNP calling pipelines, while reducing quality scores entropy.

### Patuzzi et al. *metaSPARSim: a 16S rRNA gene sequencing count data simulator*

The authors present metaSPARSim, a sparse count matrix simulator intended for usage in development of 16S rDNA-seq metagenomic data processing pipelines. metaSPARSim implements a new generative process that models the sequencing process with a Multivariate Hypergeometric distribution to effectively simulate 16S rDNA-seq count table, resembling real experimental data compositionality and sparsity.

### Torada et al. *ImaGene: a convolutional neural network to quantify natural selection from genomic data*

The authors explored the use of deep learning in evolutionary biology and implemented a program, called ImaGene, applying convolutional neural networks on population genomic data for the detection and quantification of natural selection. To detect and quantify signatures of positive selection, ImaGene implements a convolutional neural network which is trained using simulations.

### Boscaino et al. *MiRNA therapeutics based on logic circuits of biological pathways*

The authors address cancer related signaling pathways to investigate miRNA therapeutics. Their approach is based on drug discovery and miRNA therapeutics and uses a digital circuit simulation for cancer pathways. The most effective combination of drugs and miRNAs are then validated by the literature. Two different case studies on non-small cell lung cancer and melanoma are described.

### Verda et al. *Analyzing gene expression data for pediatric and adult cancer diagnosis using Logic Learning Machine and standard supervised methods*

The authors investigate the performance of the Logic Learning Machine (LLM) - an innovative method of supervised analysis capable of constructing models based on simple and intelligible rules - in classifying patients with cancer. Performance was evaluated using a set of eight publicly available gene expression databases for cancer diagnosis. The set of simple rules generated by LLM could contribute to a better understanding of cancer biology, potentially addressing therapeutic approaches.

### Ansaloni at al. *Exploratory analysis of transposable elements expression in the C. elegans early embryo*

The authors focus their research on Transposable Elements (TE) which are mobile sequences elements that make up large portions of eukaryote genomes. They analyzed TE expression among different cell types of the *Caenorhabditis elegans* early embryo asking if, where and when TE are expressed and whether their expression is correlated with genes playing a role in early embryo development.

### Spirito et al. *Impact of Polymorphic Transposable Elements on transcription in Lymphoblastoid Cell Lines from Public Data*

In this work, the authors addressed another aspect of TEs: their presence in cis on the expression of flanking genes by producing associations between polymorphic TEs and flanking gene expression levels in human lymphoblastoid cell lines. They exploit an expression quantitative trait loci approach integrated with additional bioinformatics data mining analyses.

## Data Availability

Not applicable
